# Predicted effect of ticagrelor on the pharmacokinetics of dabigatran etexilate using physiologically based pharmacokinetic modeling

**DOI:** 10.1038/s41598-020-66557-x

**Published:** 2020-06-16

**Authors:** Nan Wang, Lu Chen, Na Li, Gaoqi Xu, Fang Qi, Liqin Zhu, Wensheng Liu

**Affiliations:** 1 Pharmacy Department, Tianjin Third Central Hospital, Tianjin, China; 2 Tianjin Key Laboratory of Artificial Cell, Tianjin, China; 3 Artificial Cell Engineering Technology Research Center of Public Health Ministry, Tianjin, China; 4 Pharmaceutical College, Tianjin Medical University, Tianjin, China; 5 Pharmacy Department, Zhejiang Cancer Hospital, Hangzhou, China; 6 Pharmacy Department, Tianjin First Center Hospital, Tianjin, China

**Keywords:** Drug safety, Pharmacology

## Abstract

Dabigatran etexilate (DABE) is a direct oral anticoagulant (DOAC) and may be combined with ticagrelor, a P2Y_12_ inhibitor with antiplatelet effects. This combination of antiplatelet drugs and anticoagulants would increases the risk of bleeding in patients. In addition, the potential drug interaction may further increase the risk of bleeding. At present, there is scarce research to clarify the results of the interaction between the two. Therefore, we conducted this study to identify the potential impact of ticagrelor on the pharmacokinetics of DABE using physiologically based pharmacokinetic (PBPK) modeling. The models reasonably predicted the concentration-time profiles of dabigatran (DAB), the transformation form after DABE absorption, and ticagrelor. For pharmacokinetic drug-drug interaction (DDI), exposure to DAB at steady state was increased when co-administrated with ticagrelor. The C_max_ and AUC_0-t_ of DAB were raised by approximately 8.7% and 7.1%, respectively. Meanwhile, a stable-state ticagrelor co-administration at 400 mg once-daily increased the C_max_ and AUC_0-t_ of DAB by approximately 12.8% and 18.8%, respectively. As conclusions, Ticagrelor slightly increased the exposure of DAB. It is possible to safely use ticagrelor in a double or triple antithrombotic regimen containing DABE, only considering the antithrombotic efficacy, but not need to pay much attention on the pharmacokinetic DDI.

## Introduction

Previously referred to as new oral anticoagulants, the direct oral anticoagulants (DOACs) are a group of rapidly acting and directly clotting factors inhibiting anticoagulants. Current available DOACs include dabigatran etexilate (DABE), rivaroxaban, apixaban and edoxaban^1^. Among the DOACs, DABE is a particular Factor IIa (thrombin) inhibitor, used for (1) the prevention of stroke and systemic embolism in patients with nonvalvular atrial fibrillation (AF)^2^, (2) the treatment of acute venous thromboembolism and prevention of venous thromboembolism recurrence^3^, and (3) the prevention of venous thromboembolism after hip or knee replacement surgery^4^. DABE is a prodrug, which is converted to the active form dabigatran (DAB) after absorption. Both the prodrug and the metabolite are excreted via the renal route^5^, and not involved in the cytochrome P450 (CYP450) enzymes, regarding as an advantage over warfarin and many other DOACs. However, DABE, but not DAB, is a substrate for permeability glycoprotein (P-gp)^2^, which is an important efflux transporter protein, affecting the absorption of DABE^6^. Therefore, a P-gp inhibitor, such as amiodarone, verapamil, ketoconazole, clarithomycin, may increase the area under the plasma concentration–time curve (AUC) of DAB from about 50% to over 200%^7^. Besides, the P-gp mediated drug interactions between DABE and antibiotics, proton pump inhibitors, antiepileptic drugs or some new antiretroviral/antiproliferative drugs have also been reported^8–12^.

Ticagrelor is a novel, reversible, and direct oral adenosine diphosphate receptor P2Y_12_ inhibitor, and is applied in patients with acute coronary syndromes worldwide^13–15^. In the aspect of pharmacokinetics (PK), ticagrelor is both a P-gp substrate and a P-gp inhibitor that may affect substrates transported by the P-gp^16^.

The concomitant use of antagonist and antiplatelet agents can reduce the risk of all-cause death, myocardial infarction (MI), stroke and venous thrombosis in patients suffering from acute coronary syndrome (ACS)^17,18^, and has been extensively applied in patients with both AF and ischemic heart diseases, especially after percutaneous coronary intervention (PCI)^19,20^. As new antithrombotic drugs, DABE in combination with ticagrelor offers an additional option to substitute warfarin regimen, with the expectation of decreasing the bleeding risk and simultaneously maintaining the clinical effect^21^.

Thus, researchers have focused on the antithrombotic activation and effect of preventing cardiovascular events of DABE in conjunction with ticagrelor^22,23^, reaching conclusions that a triple therapy of DABE in combination with ticagrelor plus aspirin is as effective as warfarin triple regimen^22^, and dual therapy of DABE plus a P2Y_12_ inhibitor (clopidogrel or ticagrelor) is non-inferior to triple therapy of warfarin plus a P2Y_12_ inhibitor (clopidogrel or ticagrelor) and aspirin among patients with atrial fibrillation after PCI, and the risk of bleeding is lower^23^.

However, the potential P-gp induced drug-drug interaction (DDI) between DABE and ticagrelor, which may lead to an elevation of DAB plasma concentration, has not been accurately described. In addition, the indications for this dual therapy are still being explored. Our study aims to use a physiologically based pharmacokinetic (PBPK) model to predict PK profiles and to assess the P-gp mediated DDI of DABE when co-administered with multiple doses of ticagrelor.

## Results

### Physiologically based pharmacokinetic model and verification

We simulated blood concentration models of 150 mg dabigatran and 200 mg ticagrelor. Blood concentration data at each time point see Supplementary Table 1 and Supplementary Table 2.

The models reasonably predicted the concentration–time profiles of DAB (C_max_ 0.11 μg/mL predicted vs. 0.11 μg/mL observed, T_max_ 1.76 h predicted vs. 1.97 h observed, AUC0-t 0.69 μg·h/mL predicted vs. 0.70 μg·h/mL observed) and ticagrelor (C_max_ 0.80 μg/mL predicted vs. 0.88 μg/mL observed, T_max_ 2.28 h predicted vs. 1.92 h observed, AUC0-t 6.56 μg·h/mL predicted vs. 6.52 μg·h/mL observed).

**Figure 1:**
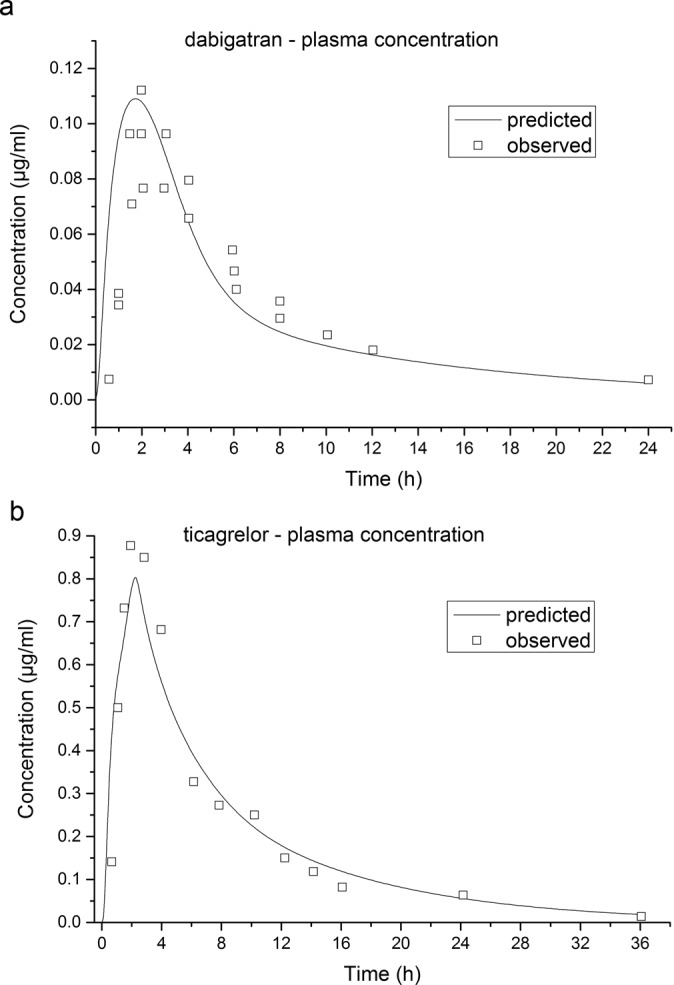
Observed (squares) and physiologically based pharmacokinetic (PBPK) model-stimulated (lines) plasma concentration-time profile of dabigatran and ticagrelor: (**a**) dabigatran 150 mg oral (**b**) ticagrelor 200 mg oral.

The *in vivo* data were loaded to verify the predictive accuracy. The simulations and verifications of the plasma concentration–time curves for DAB at a dose of DABE 150 mg and ticagrelor at a dose of 200 mg were shown in Fig. 1a,b, respectively, revealing that the simulated profiles for both DAB and ticagrelor were qualitatively similar to the observed data.

The simulated plasma concentration–time profiles of DAB and ticagrelor corresponded well with the observed data obtained from literatures^24,25^. In addition, the predicted PK parameters were reasonably consistent (<2-fold error) with the observed values, which indicated that the models were successfully and accurately simulated the pharmacokinetic process of the two medications. The predicted and observed PK parameters with prediction accuracy were summarized in Table 2.

**Table 1:** Summary of model parameters used in simulations.

Parameters	Unit	Dabigatran Etexilate	Ticagrelor
***Physicochemical parameters***			
Molecular weight	g/mol	627.73^a^	522.57^a^
Dosage form		Capsule	Tablet
logP		4.59^a^	2.28^a^
pKa (acid)		17.89^a^	12.94^a^
fu (plasma)	%	65%^a^	0.2%^a^
Aqueous solubility	mg/mL	1.8^a^	0.063^a^
R_bp_	fold	3.08^b^	0.4^b^
P_eff_	cm/s * 10^4^	1^b^	2^b^
***Metabolism data***			
P-gp V_max_		10^c^	
P-gp K_m_	μM	1.0^c^	

pKa, acid dissociation constant; P_eff_, effective permeability; R_bp_, blood/plasma concentration ratio; fu (plasma), fraction unbound in plasma; logP, partition coefficient.

^a^From DrugBank (https://www.drugbank.ca/).

^b^Estimated by ADMET Predictor.

^c^Zhao Y. *et al*. (2014).

**Table 2:** Observed and simulated pharmacokinetic parameters of ticagrelor and dabigatran etexilate.

		C_max_ (μg/mL)	T_max_ (h)	AUC_0-inf_ (μg·h/mL)	AUC_0-t_ (μg·h/mL)
Ticagrelor	Observed	0.88	1.92	6.63	6.52
	Predicted	0.80	2.28	6.76	6.56
	Fold-error	1.10	0.84	0.98	0.99
Dabigatran	Observed	0.11	1.97	0.79	0.70
	Predicted	0.11	1.76	0.76	0.69
	Fold-error	1.00	1.12	1.04	1.01

C_max_, maximum plasma concentration; T_max_, time from last dosing to the maximum plasma concentration; AUC_0-inf_, area under the concentration-time curve over the simulated period; AUC_0-t_, area under the concentration-time curve till infinite.

### Drug-drug Interaction simulation with DABE and ticagrelor

**Figure 2:**
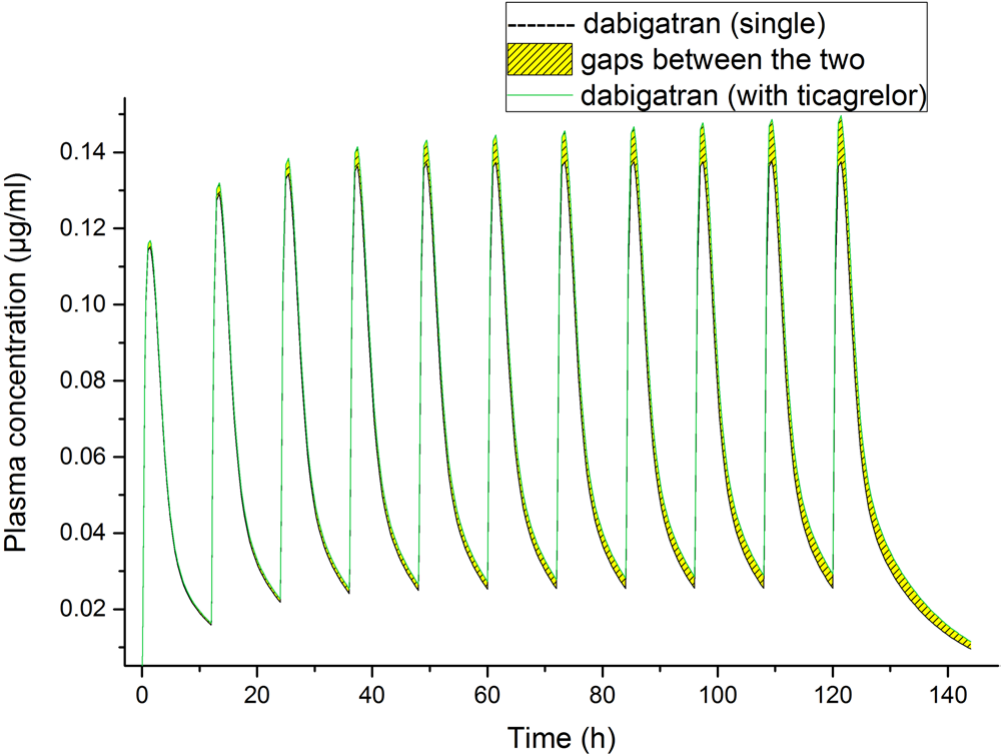
Stimulated mean plasma concentrations of dabigatran over time following multiple administrations of 150 mg twice-daily dabigatran etexilate alone or with multiple dose of ticagrelor at 90 mg twice daily.

A dynamic DDI simulation was performed to predict the effect of ticagrelor on the PK of DABE, using multiple doses in the PBPK model for 5 days (10 doses). The plasma concentration–time curves of DAB at baseline and following DDI were shown in Fig. 2, blood concentration data in Supplementary Table 3. The model-predicted ratios of DAB C_max_ and AUC_0-t_ with ticagrelor co-administration were 1.087 and 1.071 (Table 3).

**Table 3:** Simulated pharmacokinetic parameters of dabigatran following multiple administrations of 150 mg twice-daily dabigatran etexilate alone or with multiple dose of ticagrelor at 90 mg twice daily.

	C_max_ (μg/mL)	T_max_ (h)	AUC_0-t_ (μg·h/mL)	AUC_0-inf_ (μg·h/mL)
Ticagrelor-baseline/DDI	13.48	123.4	1190000	98300000
Dabigatran-baseline	0.138	121.3	8334.9	8452.9
Dabigatran-DDI	0.15	121.3	8925.4	9073.3
Dabigatran-ratio	1.087	1	1.071	1.073

C_max_, maximum plasma concentration; T_max_, time from last dosing to the maximum plasma concentration; AUC_0-inf_, area under the concentration-time curve over the simulated period; AUC_0-t_, area under the concentration-time curve till infinite; DDI, drug-drug interaction.

**Figure 3:**
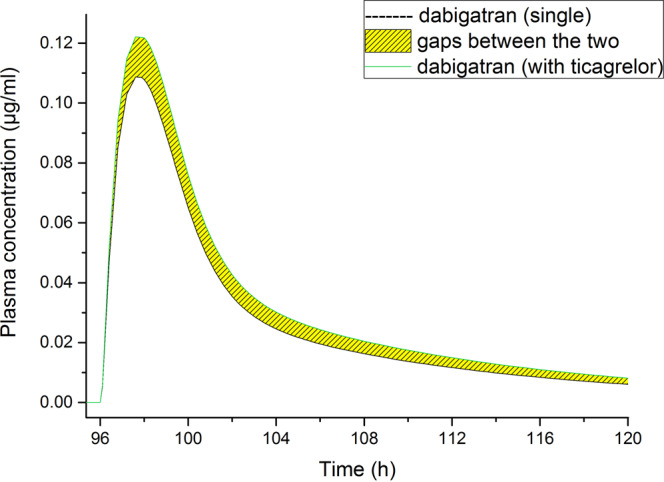
Stimulated mean plasma concentrations of dabigatran over time following multiple administrations of a single 150 mg dose dabigatran etexilate alone or with a fore-4-dose (4 days) ticagrelor at 400 mg.

In another DDI simulation, DABE were started after ticagrelor reaching stable state. The plasma concentration–time curves of DAB at baseline and following DDI after ticagrelor reaching stable state were drawn in Fig. 3, blood concentration data in Supplementary Table 4. The ratios of C_max_ and AUC_0-t_ values were ca. 1.128 and 1.188, respectively (Table 4). The result showed that the predicted ratio of DAB AUC_0-t_ was higher than the ratios observed in the former regimen.

**Table 4:** Simulated pharmacokinetic parameters of dabigatran following multiple administrations of a single 150 mg dose dabigatran etexilate alone or with a fore-4-dose (4 days) ticagrelor at 400 mg.

	C_max_ (μg/mL)	T_max_ (h)	AUC_0-t_ (μg·h/mL)	AUC_0-inf_ (μg·h/mL)
Ticagrelor-baseline/DDI	24.2	106	1700000	16100000
Dabigatran-baseline	0.109	97.7	693.4	768.2
Dabigatran-DDI	0.123	97.8	823.7	931
Dabigatran-ratio	1.128	1.001	1.188	1.212

C_max_, maximum plasma concentration; T_max_, time from last dosing to the maximum plasma concentration; AUC_0-inf_, area under the concentration-time curve over the simulated period; AUC_0-t_, area under the concentration-time curve till infinite; DDI, drug-drug interaction.

## Discussion

DABE is a new oral anticoagulant used to reduce the risk of stroke and systemic embolism in non-valvular atrial fibrillation and to treat and prevent blood clots in veins. In the RE-DEEM trial^26^, DABE was given in addition to dual antiplatelet treatment (aspirin plus clopidogrel) in patients with a recent myocardial infarction at high risk of new ischaemic cardiovascular events. Ticagrelor is deemed as an alternative to clopidogrel in patients with acute coronary syndromes, especially with clopidogrel resistance^27^. Currently, concurrent medication with ticagrelor and different anti-coagulation agents has been paid great attention^22,23,28–31^, including DABE plus ticagrelor, which was not yet recommended a few years ago^32^. As mentioned, DABE is a prodrug that has a low oral availability of around 7%, for DABE is an intestinal P-gp substrate^6^. Whereas, *in vitro* studies have indicated that ticagrelor is a substrate and inhibitor of P-gp^16^. The current study was conducted to predict the potential interaction between DABE and ticagrelor, by comparing the pharmacokinetics of DAB alone and in combination with ticagrelor using PBPK modeling.

PBPK models are proved useful to integrate all the parameters which affect the pharmacokinetics, for example, the parameters associated with the properties of the drugs and the physiological parameters specific to the animal species. There are now many softwares available (such as GastroPlus, PKsim, Simcyp) which include the parameters, and equations describing the mechanisms involved in drug disposition, metabolism and excretion^33^. PBPK modeling and simulation were performed using the Simcyp Simulator in assessing potential DDIs between DABE and a P-gp inhibitor in renal impairment populations in Doki’s study^34^. GastroPlus was used to performed the PBPK models in the prediction of ticagrelor and its active metabolite in liver cirrhosis populations in Zhang's study^35^. Both PBPK models in Zhang’s and our study were performed and simulated using GastroPlus™. Although it is slightly different from Zhang’s modeling method, we validated the reliability of the model using data from clinical literature, which prompts the results are credible.

We designed two DDI simulations. One simulation was DABE 150 mg bid Day 1–5 + ticagrelor (180 mg loading dose followed by 90 mg bid) Day 1-5. Both doses were the maximum common doses currently used in clinical. As a result, a slight increase in DAB exposure at steady state was observed when co-administrated with ticagrelor - the C_max_ and AUC_0-t_ of DAB were raised by approximately 8.7% and 7.1%, respectively. As the DDI was small, we only validated 150 mg DABE dose, and did not put lower dosage into DDI simulation. In the other simulation, we designed a DABE 150 mg single dose after 5 days continuous use of ticagrelor 400 mg qd, which was higher than the approved 90 mg bid maintenance dose used clinically. This design was based on the previous study of the interaction between ticagrelor and digoxin^36^. In this dose regimen, ticagrelor increased the C_max_ and AUC_0-t_ of DAB by approximately 12.8% and 18.8%, respectively, indicating that there was limited influence of ticagrelor on the PK parameters of DAB, even at a relatively high ticagrelor level.

In a previous study, Weisshaar et al investigated the pharmacodynamic and pharmacokinetic effect of orally administered ticagrelor and aspirin in combination with DABE in healthy male subjects, compared with DABE alone^22^. As a result, the median DAB plasma concentration was increased 130% at 3 h after concomitant use of DABE, ticagrelor and aspirin, versus single-dose DABE alone. Besides, in the summary of product characteristics of DABE on European Medicines Agency (EMA), drug interaction data for DABE-ticagrelor showed a widely varied AUC and C_max_ increase, which were higher than the change in our model prediction. We analyzed the possible reasons were as follows: First, the research population was different. The research population of our study was healthy young people with normal body weight, while the results listed in the EMA Summary of product characteristics may be the result of a combination of various population. Second, from a mechanistic perspective, ticagrelor is a weak P-gp inhibitor, which has a certain effect on drug transport, but the inhibitory effect is relatively weak. Compared with placebo, the AUCτ of digoxin, a substrate of renal and intestinal P-gp, was increased by approximately and 28 % when co-administration with ticagrelor^36^. Similarly, ticagrelor increased the C_max_ and AUC_0-inf_ of cyclosporine, another substrate of intestinal P-gp^37^, by approximately 5 % and 12 %, respectively^38^, proving ticagrelor is a weak inhibitor of intestinal P-gp. Therefore, our results were reasonable from the perspective of mechanism analysis.

With respect to efficacy, in patients with atrial fibrillation who had undergone PCI, the risk of bleeding was lower and non-inferior among those who receive dual therapy with DABE and ticagrelor (or clopidogrel), comparing to those who receive triple therapy with warfarin, ticagrelor (or clopidogrel) and aspirin, in RE-DUAL trail^23^. Regrettably, ticagrelor was not grouped separately, so that we were wondering whether the satisfactory efficacy was corresponding with the DDI between DABE and ticagrelor. Besides, further studies are in need of conducting to explore this dual therapy in the other indications for combination.

From another aspect, since both CYP3A4 and P-gp were involved in the disposition of rivaroxaban, apixaban and edoxaban^39,40^, clinical consequences may be affected when one of the three xabans was co-administrated with ticagrelor. In GEMINI-ACS-1 trial^41^, the frequency of TIMI non-CABG relevant bleeding for rivaroxaban plus ticagrelor was significantly increased compared to rivaroxaban plus clopidogrel. This result may partially blame on the DDI between rivaroxaban and ticagrelor. Therefore, it may be more secure to combine ticagrelor with DABE than other DOACs when using the routine dosage, for the pharmacokinetic changes of DABE are limited. Besides, it is possible to be a safety choice to use ticagrelor in a double or triple antithrombotic therapy with DABE.

Although the increases of DAB plasma concentration were considered unlikely to be of clinical significance, close clinical and laboratory monitoring was still highly recommended, referring to the higher rate of total bleeding at 33.3%/27.1% in patients receiving DABE 150 mg/110 mg plus ticagrelor (or clopidogrel)^23^, compared with the rate at 16.42%/14.62% with DABE 150 mg/110 mg alone^42^ or 16.1% with ticagrelor alone^13^, particularly in special populations such as elderly, renal dysfunction and other patients with high risk of bleeding. As DAB is mainly excreted by the kidneys, concomitant renal insufficiency may further increase DAB exposure when given together with P-gp inhibitors. This theoretically supports the findings of the literature, which found impaired renal function, co-medication with antiplatelet drugs or P-gp inhibitors are the risk factors for bleeding with DOACs^43^.

Overall, the results of this study fill in gaps in the effect of ticagrelor on the PK parameters of DAB, so as to provide support for the clinical application and further research of the combination of these two drugs.

There are several limitations to the current work. First, virtual Caucasian healthy male adults were simulated in the study, lacking for the evaluation on the other population like the elderly, female etc. A human study revealed that DAB exposure in female Caucasian healthy volunteers was about 25% higher than in males. This gender difference is most likely to be attributable to the lower body weight and lower creatinine clearance in females, which in turn results in lower drug clearance than males^44^. Second, the inhibitory effects of ticagrelor on other DOACs, i.e. rivaroxaban, apixaban, edoxaban, were not assessed, due to the limitation of published *in vitro* P-gp transporter study specifically designed to capture certain parameters that is required in the process of DDI predicting. Finally, the efficacy and safety of this co-administration therapy should be further verified by more clinical experience and experiments.

## Conclusions

DABE and ticagrelor showed a pharmacokinetic interaction. At steady-state, DAB C_max_ and AUC_0–t_ were increased by 8.5% and 7.1%, respectively, in the presence of ticagrelor versus DABE alone. Meanwhile, after a 4-day loading of ticagrelor, DAB C_max_ and AUC_0–t_ were increased by 12.8% and 18.8%, respectively, versus DABE alone. Since the changes in pharmacokinetic parameters are limited, it is possible to safely use ticagrelor in a double or triple antithrombotic therapy with DABE. Based on these findings, it is recommended that DABE and ticagrelor can be used concomitantly, only considering the antithrombotic efficacy, but not need to pay much attention on the pharmacokinetic DDI.

## Methods

The construction of PBPK models and the simulation of drug interaction were performed with GastroPlus v 9.0 (Simulations Plus Inc. Lancaster, CA). The models were constructed and refined to match the observed maximum plasma concentration (C_max_) and area under the plasma concentration–time curve (AUC) values from reported literatures. The Population Estimates for Age-Related (PEAR) human physiology model was used to assume that the typical study subjects were 30-year-old American healthy males weighing 78 kg.

### Structure and validation of DAB and ticagrelor PBPK models

The PBPK models were developed via known physicochemical and PK parameters, which were initially required to run simulations with GastroPlus, including: formulation; molecular weight; partition coefficient (logP); the acid dissociation constant (pKa); fraction unbound in plasma (f_up_); aqueous solubility; blood/plasma concentration ratio (R_bp_); effective permeability (P_eff_). These parameters were obtained from DrugBank (https://www.drugbank.ca/) or acquired from the ADMET Predictor (Simulations Plus Inc.) which is an inbuilt module within GastroPlus. Adjusted plasma f_up_ values were used in the models. The P-gp and kinetic inputs such as maximum reaction velocity (V_max_) and Michaelis–Menten constant (K_m_) were obtained from the published literature^24^. All the parameters were listed in Table 1.

After the base PBPK models were constructed, simulations were conducted with an initial dose of DABE 150 mg or ticagrelor 200 mg. The predicted plasma concentration–time curves were validated using data from experimental human studies where volunteers received a single dose of DABE 150 mg or ticagrelor 200 mg. The overall accuracy of the predicted PK parameters was assessed by the fold-error (difference between predicted and observed *in vivo* values), and the prediction was considered successful if the fold-error was <2^45^.

### Quantitative prediction of drug-drug interaction

The DDI between DABE and ticagrelor in virtual healthy volunteers were simulated in the PBPK model, using GastroPlus, to predict the inhibitory effect of ticagrelor on the plasma concentration–time data of DAB.

The *in vitro* inhibition constant (K_i_) of ticagrelor on human P-gp was calculated from Eq1 with published data^24,36^.

1 $${\rm{ki}}=\frac{1}{0}.4=1.\frac{5}{0}.4=3.75\,{\rm{\mu }}{\rm{m}}$$

Dynamic simulations of DAB plasma concentration–time profiles taken with and without ticagrelor were run with the DDI Module within GastroPlus. The dose and dose interval of the substrate and inhibitor were set based on the US Food and Drug Administration (FDA) drug instructions.

All virtual subjects received oral DABE 150 mg twice-daily (Day 1–5). Besides, a loading dose of 180 mg and maintenance doses of 90 mg twice-daily ticagrelor was co-administered with DABE on Day 1-5, to determine the inhibitory activity.

The DDI was further evaluated in another PBPK model, in which, ticagrelor at 400 mg once daily^36^ was delivered on days 1-4, and then, DABE at 150 mg was added to the 5th dose of ticagrelor, when ticagrelor had reached stable state.

A number of PK parameters of DABE were predicted by the DDI models. These parameters included C_max_, time from last dosing to the maximum concentration of the analyte in plasma at steady state (T_max_) and area under the concentration-time curve at steady state over the simulated period (AUC_0-t_) and till infinite (AUC_0-inf_). In addition, DAB concentration-time curves for both models were depicted.

## Supplementary information

Supporting information.
