# Supplementary material for: Predicted effect of ticagrelor on the pharmacokinetics of dabigatran etexilate using physiologically based pharmacokinetic modeling
Source: Sci Rep. 2020 Jun 16;10:9717. doi: 10.1038/s41598-020-66557-x (PMC7298054; doi:10.1038/s41598-020-66557-x)
Supplement: Supplementary file 1 — Supporting information. [file 41598_2020_66557_MOESM1_ESM.pdf]

Supplementary Table 1 Dabigatran modeling data

Simulation		Verification	
Time (h)	Dabigatran Concentration (ug/mL)	Time (h)	Dabigatran Concentration (ug/mL)
0	0	0.5773	7.46E-03
0.08	0.00349	0.9897	0.0343
0.1	0.0055	0.9897	0.0385
0.16	0.01304	1.4845	0.0963
0.24	0.02453	1.567	0.0709
0.32	0.036	1.9794	0.1121
0.4	0.04674	1.9794	0.0963
0.48	0.05649	2.0619	0.0766
0.56	0.06518	2.9691	0.0766
0.5773	0.06692	3.0516	0.0963
0.64	0.07282	4.0412	0.0795
0.72	0.07947	4.0412	0.0657
0.8	0.0852	5.9381	0.0543
0.88	0.09009	6.0206	0.0466
0.96	0.09422	6.1031	0.04
0.9897	0.09558	8	0.0295
1.04	0.09769	8	0.0357
1.12	0.10057	10.0619	0.0235
1.2	0.10293	12.0412	0.018
1.28	0.10482	24	7.18E-03
1.36	0.10631		
1.44	0.10744		
1.4845	0.10793		
1.52	0.10825		
1.567	0.10859		
1.6	0.10877		
1.68	0.10902		
1.76	0.10903		
1.84	0.10882		
1.92	0.10841		
1.9794	0.10798		
2	0.10781		
2.0619	0.10722		
2.08	0.10703		
2.16	0.10609		
2.24	0.105		
2.32	0.10377		
2.4	0.10242		

2.48	0.10095		
2.56	0.09938		
2.64	0.09771		
2.72	0.09597		
2.8	0.09416		
2.88	0.09228		
2.96	0.09037		
2.9691	0.09014		
3.04	0.08841		
3.0516	0.08812		
3.12	0.08643		
3.2	0.08443		
3.28	0.08242		
3.36	0.0804		
3.44	0.0784		
3.52	0.07641		
3.6	0.07444		
3.68	0.07249		
3.76	0.07058		
3.84	0.0687		
3.92	0.06686		
4	0.06506		
4.0412	0.06415		
4.08	0.06331		
4.16	0.0616		
4.24	0.05994		
4.32	0.05833		
4.4	0.05677		
4.48	0.05527		
4.56	0.05381		
4.64	0.0524		
4.72	0.05105		
4.8	0.04974		
4.88	0.04849		
4.96	0.04728		
5.04	0.04612		
5.12	0.04501		
5.2	0.04394		
5.28	0.04292		
5.36	0.04193		
5.44	0.04099		
5.52	0.04009		
5.6	0.03922		

5.68	0.03839		
5.76	0.0376		
5.84	0.03684		
5.92	0.03611		
5.9381	0.03595		
6	0.03541		
6.0206	0.03524		
6.08	0.03474		
6.1031	0.03456		
6.16	0.0341		
6.24	0.03349		
6.32	0.0329		
6.4	0.03233		
6.48	0.03179		
6.56	0.03127		
6.64	0.03077		
6.72	0.03029		
6.8	0.02982		
6.88	0.02938		
6.96	0.02895		
7.04	0.02854		
7.12	0.02814		
7.2	0.02776		
7.28	0.02739		
7.36	0.02703		
7.44	0.02669		
7.52	0.02636		
7.6	0.02604		
7.68	0.02573		
7.76	0.02543		
7.84	0.02513		
7.92	0.02485		
8	0.02458		
8.08	0.02431		
8.16	0.02405		
8.24	0.0238		
8.32	0.02356		
8.4	0.02332		
8.48	0.02309		
8.56	0.02286		
8.64	0.02264		
8.72	0.02243		
8.8	0.02222		

8.88	0.02202		
8.96	0.02182		
9.04	0.02162		
9.12	0.02143		
9.2	0.02124		
9.28	0.02106		
9.36	0.02088		
9.44	0.0207		
9.52	0.02053		
9.6	0.02036		
9.68	0.02019		
9.76	0.02003		
9.84	0.01987		
9.92	0.01971		
10	0.01955		
10.0619	0.01943		
10.08	0.0194		
10.16	0.01925		
10.24	0.0191		
10.32	0.01895		
10.4	0.01881		
10.48	0.01867		
10.56	0.01853		
10.64	0.01839		
10.72	0.01825		
10.8	0.01812		
10.88	0.01799		
10.96	0.01785		
11.04	0.01772		
11.12	0.0176		
11.2	0.01747		
11.28	0.01734		
11.36	0.01722		
11.44	0.0171		
11.52	0.01698		
11.6	0.01686		
11.68	0.01674		
11.76	0.01662		
11.84	0.01651		
11.92	0.01639		
12	0.01628		
12.0412	0.01622		
12.08	0.01616		

12.16	0.01605		
12.24	0.01594		
12.32	0.01583		
12.4	0.01572		
12.48	0.01562		
12.56	0.01551		
12.64	0.0154		
12.72	0.0153		
12.8	0.0152		
12.88	0.01509		
12.96	0.01499		
13.04	0.01489		
13.12	0.01479		
13.2	0.01469		
13.28	0.01459		
13.36	0.01449		
13.44	0.0144		
13.52	0.0143		
13.6	0.0142		
13.68	0.01411		
13.76	0.01401		
13.84	0.01392		
13.92	0.01383		
14	0.01374		
14.08	0.01365		
14.16	0.01355		
14.24	0.01346		
14.32	0.01338		
14.4	0.01329		
14.48	0.0132		
14.56	0.01311		
14.64	0.01303		
14.72	0.01294		
14.8	0.01285		
14.88	0.01277		
14.96	0.01268		
15.04	0.0126		
15.12	0.01252		
15.2	0.01244		
15.28	0.01235		
15.36	0.01227		
15.44	0.01219		
15.52	0.01211		

15.6	0.01203		
15.68	0.01195		
15.76	0.01188		
15.84	0.0118		
15.92	0.01172		
16	0.01164		
16.08	0.01157		
16.16	0.01149		
16.24	0.01142		
16.32	0.01134		
16.4	0.01127		
16.48	0.01119		
16.56	0.01112		
16.64	0.01105		
16.72	0.01098		
16.8	0.0109		
16.88	0.01083		
16.96	0.01076		
17.04	0.01069		
17.12	0.01062		
17.2	0.01055		
17.28	0.01048		
17.36	0.01042		
17.44	0.01035		
17.52	0.01028		
17.6	0.01021		
17.68	0.01015		
17.76	0.01008		
17.84	0.01001		
17.92	0.00995		
18	0.00988		
18.08	0.00982		
18.16	0.00976		
18.24	0.00969		
18.32	0.00963		
18.4	0.00957		
18.48	0.0095		
18.56	0.00944		
18.64	0.00938		
18.72	0.00932		
18.8	0.00926		
18.88	0.0092		
18.96	0.00914		

19.04	0.00908		
19.12	0.00902		
19.2	0.00896		
19.28	0.0089		
19.36	0.00885		
19.44	0.00879		
19.52	0.00873		
19.6	0.00868		
19.68	0.00862		
19.76	0.00856		
19.84	0.00851		
19.92	0.00845		
20	0.0084		
20.08	0.00834		
20.16	0.00829		
20.24	0.00824		
20.32	0.00818		
20.4	0.00813		
20.48	0.00808		
20.56	0.00802		
20.64	0.00797		
20.72	0.00792		
20.8	0.00787		
20.88	0.00782		
20.96	0.00777		
21.04	0.00772		
21.12	0.00767		
21.2	0.00762		
21.28	0.00757		
21.36	0.00752		
21.44	0.00747		
21.52	0.00742		
21.6	0.00737		
21.68	0.00733		
21.76	0.00728		
21.84	0.00723		
21.92	0.00718		
22	0.00714		
22.08	0.00709		
22.16	0.00705		
22.24	0.007		
22.32	0.00695		
22.4	0.00691		

22.48	0.00686		
22.56	0.00682		
22.64	0.00678		
22.72	0.00673		
22.8	0.00669		
22.88	0.00665		
22.96	0.0066		
23.04	0.00656		
23.12	0.00652		
23.2	0.00647		
23.28	0.00643		
23.36	0.00639		
23.44	0.00635		
23.52	0.00631		
23.6	0.00627		
23.68	0.00623		
23.76	0.00619		
23.84	0.00615		
23.92	0.00611		
24	0.00607		

Supplementary Table 2 Ticagrelor modeling data

Simulation		Verification	
Time (h)	Ticagrelor Concentration (ug/mL)	Time (h)	Ticagrelor Concentration (ug/mL)
0	0	0.6586	0.1409
0.1	0.0102	1.0679	0.5
0.12	0.01791	1.4915	0.7318
0.2	0.06859	1.9248	0.8773
0.24	0.10192	2.8272	0.85
0.3	0.15671	3.9702	0.6818
0.36	0.2137	6.1459	0.3273
0.4	0.25135	7.8382	0.2727
0.48	0.32054	10.2014	0.25
0.5	0.33604	12.2361	0.15
0.6	0.40299	14.1506	0.1182
0.6586	0.43511	16.0657	0.0818
0.72	0.46423	24.1615	0.0636
0.7586	0.48056	36.0828	0.0136
0.84	0.51093		
0.8586	0.51722		
0.94	0.54238		
0.96	0.54806		
1.04	0.56916		
1.0679	0.57595		
1.08	0.57882		
1.14	0.59227		
1.2	0.60457		
1.24	0.61232		
1.32	0.62836		
1.34	0.63256		
1.44	0.65441		
1.4915	0.666		
1.56	0.6816		
1.5915	0.6888		
1.68	0.70926		
1.6915	0.71202		
1.78	0.73385		
1.8	0.73879		
1.88	0.75751		
1.92	0.76578		
1.9248	0.76671		
1.98	0.77673		

2.04	0.78625		
2.08	0.79182		
2.16	0.80046		
2.18	0.80193		
2.28	0.80283		
2.38	0.79247		
2.4	0.78952		
2.52	0.76931		
2.64	0.74768		
2.76	0.72635		
2.8272	0.71476		
2.88	0.70588		
3	0.68645		
3.12	0.66808		
3.24	0.65072		
3.36	0.6343		
3.48	0.61871		
3.6	0.60387		
3.72	0.58969		
3.84	0.57611		
3.96	0.56307		
3.9702	0.56199		
4.08	0.55052		
4.2	0.53841		
4.32	0.52672		
4.44	0.51543		
4.56	0.5045		
4.68	0.49393		
4.8	0.4837		
4.92	0.4738		
5.04	0.4642		
5.16	0.45491		
5.28	0.44591		
5.4	0.43718		
5.52	0.42872		
5.64	0.42051		
5.76	0.41254		
5.88	0.4048		
6	0.39727		
6.12	0.38995		
6.1459	0.38839		
6.24	0.38282		
6.36	0.37587		

6.48	0.3691		
6.6	0.3625		
6.72	0.35605		
6.84	0.34976		
6.96	0.34361		
7.08	0.3376		
7.2	0.33173		
7.32	0.32599		
7.44	0.32037		
7.56	0.31488		
7.68	0.30951		
7.8	0.30425		
7.8382	0.30261		
7.92	0.29912		
8.04	0.29409		
8.16	0.28918		
8.28	0.28438		
8.4	0.27968		
8.52	0.2751		
8.64	0.27061		
8.76	0.26623		
8.88	0.26195		
9	0.25777		
9.12	0.25368		
9.24	0.24969		
9.36	0.24579		
9.48	0.24199		
9.6	0.23827		
9.72	0.23464		
9.84	0.23109		
9.96	0.22762		
10.08	0.22423		
10.2	0.22093		
10.2014	0.22089		
10.32	0.21769		
10.44	0.21453		
10.56	0.21145		
10.68	0.20843		
10.8	0.20547		
10.92	0.20259		
11.04	0.19976		
11.16	0.197		
11.28	0.19429		

11.4	0.19165		
11.52	0.18905		
11.64	0.18651		
11.76	0.18403		
11.88	0.18159		
12	0.1792		
12.12	0.17686		
12.2361	0.17464		
12.24	0.17457		
12.36	0.17231		
12.48	0.1701		
12.6	0.16794		
12.72	0.16581		
12.84	0.16372		
12.96	0.16167		
13.08	0.15965		
13.2	0.15767		
13.32	0.15572		
13.44	0.15381		
13.56	0.15193		
13.68	0.15008		
13.8	0.14826		
13.92	0.14647		
14.04	0.14471		
14.1506	0.14311		
14.16	0.14297		
14.28	0.14126		
14.4	0.13958		
14.52	0.13793		
14.64	0.1363		
14.76	0.13469		
14.88	0.13311		
15	0.13155		
15.12	0.13001		
15.24	0.1285		
15.36	0.127		
15.48	0.12553		
15.6	0.12408		
15.72	0.12264		
15.84	0.12123		
15.96	0.11984		
16.0657	0.11862		
16.08	0.11846		

16.2	0.1171		
16.32	0.11577		
16.44	0.11444		
16.56	0.11314		
16.68	0.11185		
16.8	0.11058		
16.92	0.10932		
17.04	0.10809		
17.16	0.10686		
17.28	0.10565		
17.4	0.10446		
17.52	0.10328		
17.64	0.10212		
17.76	0.10097		
17.88	0.09983		
18	0.09871		
18.12	0.0976		
18.24	0.09651		
18.36	0.09542		
18.48	0.09435		
18.6	0.0933		
18.72	0.09225		
18.84	0.09122		
18.96	0.0902		
19.08	0.0892		
19.2	0.0882		
19.32	0.08722		
19.44	0.08624		
19.56	0.08528		
19.68	0.08433		
19.8	0.08339		
19.92	0.08247		
20.04	0.08155		
20.16	0.08064		
20.28	0.07975		
20.4	0.07886		
20.52	0.07798		
20.64	0.07712		
20.76	0.07626		
20.88	0.07541		
21	0.07458		
21.12	0.07375		
21.24	0.07293		

21.36	0.07212		
21.48	0.07132		
21.6	0.07053		
21.72	0.06975		
21.84	0.06898		
21.96	0.06822		
22.08	0.06746		
22.2	0.06671		
22.32	0.06597		
22.44	0.06524		
22.56	0.06452		
22.68	0.06381		
22.8	0.0631		
22.92	0.0624		
23.04	0.06171		
23.16	0.06103		
23.28	0.06036		
23.4	0.05969		
23.52	0.05903		
23.64	0.05838		
23.76	0.05773		
23.88	0.05709		
24	0.05646		
24.12	0.05584		
24.1615	0.05562		
24.24	0.05522		
24.36	0.05461		
24.48	0.05401		
24.6	0.05341		
24.72	0.05282		
24.84	0.05224		
24.96	0.05166		
25.08	0.05109		
25.2	0.05052		
25.32	0.04997		
25.44	0.04941		
25.56	0.04887		
25.68	0.04833		
25.8	0.0478		
25.92	0.04727		
26.04	0.04675		
26.16	0.04623		
26.28	0.04572		

26.4	0.04521		
26.52	0.04472		
26.64	0.04422		
26.76	0.04373		
26.88	0.04325		
27	0.04277		
27.12	0.0423		
27.24	0.04183		
27.36	0.04137		
27.48	0.04092		
27.6	0.04046		
27.72	0.04002		
27.84	0.03958		
27.96	0.03914		
28.08	0.03871		
28.2	0.03828		
28.32	0.03786		
28.44	0.03744		
28.56	0.03703		
28.68	0.03662		
28.8	0.03621		
28.92	0.03581		
29.04	0.03542		
29.16	0.03503		
29.28	0.03464		
29.4	0.03426		
29.52	0.03388		
29.64	0.03351		
29.76	0.03314		
29.88	0.03277		
30	0.03241		
30.12	0.03205		
30.24	0.0317		
30.36	0.03135		
30.48	0.031		
30.6	0.03066		
30.72	0.03032		
30.84	0.02999		
30.96	0.02966		
31.08	0.02933		
31.2	0.02901		
31.32	0.02869		
31.44	0.02837		

31.56	0.02806		
31.68	0.02775		
31.8	0.02744		
31.92	0.02714		
32.04	0.02684		
32.16	0.02654		
32.28	0.02625		
32.4	0.02596		
32.52	0.02567		
32.64	0.02539		
32.76	0.02511		
32.88	0.02483		
33	0.02456		
33.12	0.02429		
33.24	0.02402		
33.36	0.02376		
33.48	0.02349		
33.6	0.02324		
33.72	0.02298		
33.84	0.02273		
33.96	0.02247		
34.08	0.02223		
34.2	0.02198		
34.32	0.02174		
34.44	0.0215		
34.56	0.02126		
34.68	0.02103		
34.8	0.0208		
34.92	0.02057		
35.04	0.02034		
35.16	0.02012		
35.28	0.01989		
35.4	0.01967		
35.52	0.01946		
35.64	0.01924		
35.76	0.01903		
35.88	0.01882		
36	0.01861		

Supplementary Table 3 Simulated pharmacokinetic parameters of dabigatran following multiple administrations of 150mg twice-daily dabigatran etexilate alone or with multiple dose of ticagrelor at 90mg twice daily

Dabigatran etexilate alone		Dabigatran etexilate with multiple dose of ticagrelor	
Time (h)	Dabigatran Concentration (ug/mL)	Time (h)	Dabigatran Concentration (ug/mL)
0	0	0	0
0.1	0.01015	0.1	0.01016
0.48	0.08329	0.48	0.08415
0.5773	0.09369	0.5773	0.09475
0.6586	0.10021	0.6586	0.1014
0.96	0.11277	0.96	0.11427
0.9897	0.11334	0.9897	0.11487
1.0679	0.11446	1.0679	0.11606
1.44	0.11496	1.44	0.11683
1.4845	0.11466	1.4845	0.11656
1.4915	0.11461	1.4915	0.11651
1.567	0.11399	1.567	0.11591
1.5845	0.11382	1.5845	0.11575
1.5915	0.11375	1.5915	0.11569
1.667	0.11295	1.667	0.1149
1.6845	0.11274	1.6845	0.1147
1.6915	0.11266	1.6915	0.11462
1.767	0.11169	1.767	0.11366
1.7845	0.11145	1.7845	0.11341
1.7915	0.11135	1.7915	0.11332
1.867	0.11023	1.867	0.11219
1.92	0.10938	1.92	0.11133
1.9248	0.1093	1.9248	0.11125
1.9794	0.10837	1.9794	0.1103
2.0619	0.10685	2.0619	0.10878
2.0794	0.10652	2.0794	0.10844
2.1619	0.10487	2.1619	0.10677
2.1794	0.10451	2.1794	0.1064
2.2619	0.10273	2.2619	0.1046
2.2794	0.10234	2.2794	0.10421
2.3619	0.10046	2.3619	0.1023
2.4	0.09956	2.4	0.10139
2.4619	0.09806	2.4619	0.09988
2.5	0.09712	2.5	0.09893
2.5619	0.09557	2.5619	0.09735

2.6	0.09459	2.6	0.09636
2.6619	0.09299	2.6619	0.09474
2.7	0.09199	2.7	0.09373
2.7619	0.09035	2.7619	0.09207
2.8272	0.08861	2.8272	0.0903
2.88	0.08719	2.88	0.08886
2.9691	0.08478	2.9691	0.08642
3.0516	0.08255	3.0516	0.08416
3.0691	0.08207	3.0691	0.08368
3.1516	0.07985	3.1516	0.08143
3.1691	0.07939	3.1691	0.08095
3.2516	0.07719	3.2516	0.07873
3.2691	0.07673	3.2691	0.07826
3.36	0.07435	3.36	0.07584
3.3691	0.07411	3.3691	0.0756
3.46	0.07178	3.46	0.07324
3.4691	0.07155	3.4691	0.07301
3.56	0.06928	3.56	0.0707
3.5691	0.06906	3.5691	0.07047
3.66	0.06685	3.66	0.06824
3.6691	0.06664	3.6691	0.06802
3.76	0.0645	3.76	0.06585
3.84	0.06268	3.84	0.064
3.86	0.06224	3.86	0.06355
3.94	0.06049	3.94	0.06177
3.9702	0.05984	3.9702	0.06112
4.0412	0.05835	4.0412	0.05961
4.0702	0.05776	4.0702	0.059
4.1412	0.05634	4.1412	0.05756
4.1702	0.05577	4.1702	0.05698
4.2412	0.05441	4.2412	0.0556
4.32	0.05296	4.32	0.05413
4.3412	0.05258	4.3412	0.05374
4.42	0.0512	4.42	0.05233
4.4412	0.05083	4.4412	0.05196
4.52	0.04952	4.52	0.05063
4.5412	0.04917	4.5412	0.05028
4.62	0.04793	4.62	0.04901
4.6412	0.0476	4.6412	0.04868
4.72	0.04642	4.72	0.04747
4.8	0.04527	4.8	0.0463
4.82	0.04499	4.82	0.04602
4.9	0.0439	4.9	0.04491

4.92	0.04363	4.92	0.04464
5	0.0426	5	0.04359
5.28	0.03934	5.28	0.04027
5.76	0.03481	5.76	0.03566
5.9381	0.03341	5.9381	0.03423
6.0206	0.0328	6.0206	0.03362
6.1031	0.03223	6.1031	0.03303
6.1459	0.03194	6.1459	0.03273
6.24	0.03132	6.24	0.0321
6.72	0.0286	6.72	0.02933
7.2	0.02643	7.2	0.02711
7.68	0.02466	7.68	0.0253
7.8382	0.02414	7.8382	0.02478
8	0.02364	8	0.02427
8.16	0.02318	8.16	0.02379
8.64	0.0219	8.64	0.02249
9.12	0.02079	9.12	0.02135
9.6	0.0198	9.6	0.02034
10.0619	0.01893	10.0619	0.01945
10.08	0.0189	10.08	0.01942
10.2014	0.01868	10.2014	0.0192
10.56	0.01807	10.56	0.01857
11.04	0.01731	11.04	0.01779
11.52	0.01659	11.52	0.01705
12	0.01591	12	0.01636
12.0412	0.01726	12.0412	0.01772
12.1	0.02593	12.1	0.02647
12.2361	0.0566	12.2361	0.05756
12.48	0.09857	12.48	0.10041
12.96	0.12744	12.96	0.13021
13.44	0.12905	13.44	0.13205
13.46	0.1289	13.46	0.13191
13.54	0.1282	13.54	0.13121
13.56	0.128	13.56	0.13101
13.64	0.12711	13.64	0.13012
13.66	0.12686	13.66	0.12987
13.74	0.12579	13.74	0.12879
13.76	0.1255	13.76	0.1285
13.84	0.12427	13.84	0.12726
13.92	0.12292	13.92	0.12589
13.94	0.12256	13.94	0.12553
14.02	0.12106	14.02	0.12402
14.04	0.12067	14.04	0.12362

14.12	0.11904	14.12	0.12196
14.1506	0.11838	14.1506	0.1213
14.22	0.11685	14.22	0.11975
14.2506	0.11615	14.2506	0.11904
14.32	0.11452	14.32	0.11739
14.4	0.11257	14.4	0.1154
14.42	0.11207	14.42	0.1149
14.5	0.11002	14.5	0.11282
14.52	0.1095	14.52	0.11229
14.6	0.10739	14.6	0.11014
14.62	0.10685	14.62	0.1096
14.7	0.10468	14.7	0.10739
14.72	0.10413	14.72	0.10683
14.8	0.10192	14.8	0.10458
14.88	0.09969	14.88	0.10231
14.9	0.09913	14.9	0.10174
14.98	0.09688	14.98	0.09946
15	0.09632	15	0.09889
15.08	0.09408	15.08	0.0966
15.1	0.09352	15.1	0.09603
15.18	0.09129	15.18	0.09376
15.2	0.09074	15.2	0.0932
15.28	0.08854	15.28	0.09096
15.36	0.08637	15.36	0.08875
15.38	0.08583	15.38	0.0882
15.46	0.0837	15.46	0.08603
15.84	0.07424	15.84	0.07638
16.0657	0.06919	16.0657	0.07123
16.32	0.06407	16.32	0.066
16.8	0.05595	16.8	0.0577
17.28	0.04961	17.28	0.05121
17.76	0.04468	17.76	0.04617
18.24	0.04081	18.24	0.04221
18.72	0.03773	18.72	0.03904
19.2	0.03521	19.2	0.03645
19.68	0.0331	19.68	0.03429
20.16	0.03129	20.16	0.03243
20.64	0.02971	20.64	0.0308
21.12	0.0283	21.12	0.02935
21.6	0.02702	21.6	0.02804
22.08	0.02585	22.08	0.02683
22.56	0.02475	22.56	0.0257
23.04	0.02373	23.04	0.02465

23.52	0.02277	23.52	0.02366
24	0.02186	24	0.02272
24.1	0.03182	24.1	0.03282
24.1615	0.0453	24.1615	0.04652
24.48	0.10428	24.48	0.10695
24.96	0.13294	24.96	0.13674
25.44	0.13433	25.44	0.13842
25.46	0.13418	25.46	0.13826
25.54	0.13345	25.54	0.13754
25.56	0.13324	25.56	0.13733
25.64	0.13231	25.64	0.1364
25.66	0.13206	25.66	0.13614
25.74	0.13095	25.74	0.13503
25.76	0.13066	25.76	0.13473
25.84	0.12939	25.84	0.13345
25.92	0.12801	25.92	0.13205
25.94	0.12764	25.94	0.13168
26.02	0.12611	26.02	0.13013
26.04	0.12571	26.04	0.12972
26.12	0.12404	26.12	0.12803
26.14	0.12361	26.14	0.12759
26.22	0.12182	26.22	0.12577
26.24	0.12135	26.24	0.12529
26.32	0.11945	26.32	0.12335
26.4	0.11746	26.4	0.12133
26.42	0.11695	26.42	0.12081
26.5	0.11488	26.5	0.1187
26.52	0.11435	26.52	0.11816
26.6	0.1122	26.6	0.11597
26.62	0.11166	26.62	0.11541
26.7	0.10946	26.7	0.11317
26.72	0.1089	26.72	0.1126
26.8	0.10666	26.8	0.11031
26.88	0.1044	26.88	0.10799
26.9	0.10383	26.9	0.10741
26.98	0.10155	26.98	0.10509
27	0.10098	27	0.1045
27.08	0.09871	27.08	0.10218
27.1	0.09814	27.1	0.1016
27.18	0.09589	27.18	0.09929
27.2	0.09532	27.2	0.09871
27.28	0.09309	27.28	0.09643
27.36	0.09089	27.36	0.09418

27.38	0.09035	27.38	0.09362
27.46	0.08819	27.46	0.09141
27.48	0.08766	27.48	0.09087
27.84	0.07859	27.84	0.08157
28.32	0.06826	28.32	0.07096
28.8	0.05997	28.8	0.06245
29.28	0.05348	29.28	0.05576
29.76	0.04841	29.76	0.05053
30.24	0.0444	30.24	0.0464
30.72	0.04118	30.72	0.04307
31.2	0.03853	31.2	0.04032
31.68	0.03629	31.68	0.03801
32.16	0.03436	32.16	0.03601
32.64	0.03266	32.64	0.03425
33.12	0.03114	33.12	0.03267
33.6	0.02975	33.6	0.03123
34.08	0.02847	34.08	0.0299
34.56	0.02728	34.56	0.02866
35.04	0.02616	35.04	0.0275
35.52	0.02511	35.52	0.02641
36	0.02411	36	0.02537
36.0828	0.03088	36.0828	0.03226
36.1	0.03406	36.1	0.03549
36.48	0.10645	36.48	0.10993
36.96	0.13502	36.96	0.13983
37.44	0.13634	37.44	0.14148
37.46	0.13618	37.46	0.14132
37.54	0.13543	37.54	0.14058
37.56	0.13522	37.56	0.14037
37.64	0.13428	37.64	0.13943
37.66	0.13402	37.66	0.13917
37.74	0.13291	37.74	0.13804
37.76	0.13261	37.76	0.13774
37.84	0.13133	37.84	0.13645
37.92	0.12993	37.92	0.13503
37.94	0.12956	37.94	0.13465
38.02	0.12802	38.02	0.13308
38.04	0.12762	38.04	0.13267
38.12	0.12594	38.12	0.13096
38.14	0.1255	38.14	0.13051
38.22	0.1237	38.22	0.12867
38.24	0.12323	38.24	0.1282
38.32	0.12131	38.32	0.12624

38.4	0.11931	38.4	0.12419
38.42	0.1188	38.42	0.12367
38.5	0.11672	38.5	0.12154
38.52	0.11619	38.52	0.12099
38.6	0.11403	38.6	0.11878
38.62	0.11348	38.62	0.11822
38.7	0.11127	38.7	0.11595
38.72	0.11071	38.72	0.11538
38.8	0.10845	38.8	0.11306
38.88	0.10618	38.88	0.11073
38.9	0.10561	38.9	0.11014
38.98	0.10332	38.98	0.10779
39	0.10275	39	0.1072
39.08	0.10046	39.08	0.10486
39.1	0.09989	39.1	0.10427
39.18	0.09762	39.18	0.10194
39.2	0.09706	39.2	0.10136
39.28	0.09482	39.28	0.09905
39.36	0.09261	39.36	0.09677
39.38	0.09206	39.38	0.09621
39.46	0.08989	39.46	0.09398
39.48	0.08936	39.48	0.09343
39.84	0.08024	39.84	0.08403
40.32	0.06984	40.32	0.0733
40.8	0.0615	40.8	0.06467
41.28	0.05495	41.28	0.05788
41.76	0.04982	41.76	0.05256
42.24	0.04576	42.24	0.04834
42.72	0.04248	42.72	0.04493
43.2	0.03978	43.2	0.04211
43.68	0.0375	43.68	0.03973
44.16	0.03553	44.16	0.03767
44.64	0.03378	44.64	0.03584
45.12	0.03222	45.12	0.03421
45.6	0.03079	45.6	0.03271
46.08	0.02947	46.08	0.03133
46.56	0.02824	46.56	0.03004
47.04	0.02708	47.04	0.02883
47.52	0.02599	47.52	0.02768
48	0.02496	48	0.0266
48.1	0.0349	48.1	0.03676
48.48	0.10727	48.48	0.11155
48.96	0.13581	48.96	0.1416

49.44	0.1371	49.44	0.14327
49.46	0.13694	49.46	0.14311
49.54	0.13619	49.54	0.14236
49.56	0.13597	49.56	0.14215
49.64	0.13503	49.64	0.1412
49.66	0.13477	49.66	0.14094
49.74	0.13365	49.74	0.13981
49.76	0.13335	49.76	0.13951
49.84	0.13207	49.84	0.13821
49.92	0.13066	49.92	0.13678
49.94	0.13029	49.94	0.1364
50.02	0.12874	50.02	0.13482
50.04	0.12834	50.04	0.13441
50.12	0.12665	50.12	0.13269
50.14	0.12622	50.14	0.13224
50.22	0.12441	50.22	0.13039
50.24	0.12394	50.24	0.12991
50.32	0.12202	50.32	0.12794
50.4	0.12002	50.4	0.12588
50.42	0.1195	50.42	0.12536
50.5	0.11741	50.5	0.12321
50.52	0.11688	50.52	0.12266
50.6	0.11472	50.6	0.12044
50.62	0.11417	50.62	0.11987
50.7	0.11195	50.7	0.11759
50.72	0.11139	50.72	0.11701
50.8	0.10913	50.8	0.11468
50.88	0.10685	50.88	0.11233
50.9	0.10628	50.9	0.11174
50.98	0.10399	50.98	0.10938
51	0.10342	51	0.10878
51.08	0.10113	51.08	0.10642
51.1	0.10056	51.1	0.10583
51.18	0.09828	51.18	0.10348
51.2	0.09772	51.2	0.1029
51.28	0.09547	51.28	0.10058
51.36	0.09326	51.36	0.09828
51.38	0.09271	51.38	0.09772
51.46	0.09054	51.46	0.09547
51.48	0.09	51.48	0.09491
51.84	0.08086	51.84	0.08545
52.32	0.07044	52.32	0.07463
52.8	0.06208	52.8	0.06593

53.28	0.05551	53.28	0.05907
53.76	0.05035	53.76	0.05369
54.24	0.04627	54.24	0.04942
54.72	0.04298	54.72	0.04596
55.2	0.04026	55.2	0.0431
55.68	0.03796	55.68	0.04068
56.16	0.03597	56.16	0.03858
56.64	0.03421	56.64	0.03673
57.12	0.03262	57.12	0.03506
57.6	0.03118	57.6	0.03353
58.08	0.02985	58.08	0.03212
58.56	0.0286	58.56	0.03081
59.04	0.02743	59.04	0.02957
59.52	0.02633	59.52	0.0284
60	0.02528	60	0.02729
60.1	0.03522	60.1	0.03749
60.48	0.10758	60.48	0.11263
60.96	0.13611	60.96	0.14287
61.44	0.13738	61.44	0.14457
61.46	0.13722	61.46	0.14441
61.54	0.13647	61.54	0.14367
61.56	0.13626	61.56	0.14345
61.64	0.13531	61.64	0.1425
61.66	0.13505	61.66	0.14224
61.74	0.13393	61.74	0.14111
61.76	0.13363	61.76	0.1408
61.84	0.13234	61.84	0.1395
61.92	0.13094	61.92	0.13806
61.94	0.13057	61.94	0.13769
62.02	0.12902	62.02	0.1361
62.04	0.12861	62.04	0.13569
62.12	0.12693	62.12	0.13396
62.14	0.12649	62.14	0.13351
62.22	0.12468	62.22	0.13165
62.24	0.12421	62.24	0.13117
62.32	0.12229	62.32	0.12919
62.4	0.12028	62.4	0.12712
62.42	0.11977	62.42	0.12659
62.5	0.11768	62.5	0.12443
62.52	0.11714	62.52	0.12388
62.6	0.11498	62.6	0.12165
62.62	0.11443	62.62	0.12108
62.7	0.11221	62.7	0.11878

62.72	0.11165	62.72	0.1182
62.8	0.10939	62.8	0.11586
62.88	0.10711	62.88	0.1135
62.9	0.10654	62.9	0.11291
62.98	0.10424	62.98	0.11053
63	0.10367	63	0.10994
63.08	0.10138	63.08	0.10756
63.1	0.10081	63.1	0.10697
63.18	0.09853	63.18	0.1046
63.2	0.09797	63.2	0.10402
63.28	0.09572	63.28	0.10168
63.36	0.0935	63.36	0.09938
63.38	0.09295	63.38	0.09881
63.46	0.09078	63.46	0.09655
63.48	0.09024	63.48	0.09599
63.84	0.0811	63.84	0.08646
64.32	0.07067	64.32	0.07558
64.8	0.0623	64.8	0.06681
65.28	0.05572	65.28	0.05991
65.76	0.05056	65.76	0.05448
66.24	0.04647	66.24	0.05017
66.72	0.04317	66.72	0.04668
67.2	0.04044	67.2	0.04379
67.68	0.03813	67.68	0.04134
68.16	0.03613	68.16	0.03922
68.64	0.03437	68.64	0.03734
69.12	0.03278	69.12	0.03565
69.6	0.03133	69.6	0.03411
70.08	0.02999	70.08	0.03268
70.56	0.02874	70.56	0.03134
71.04	0.02756	71.04	0.03009
71.52	0.02646	71.52	0.0289
72	0.0254	72	0.02778
72.1	0.03534	72.1	0.03802
72.48	0.1077	72.48	0.11352
72.96	0.13622	72.96	0.14394
73.44	0.13749	73.44	0.14568
73.46	0.13733	73.46	0.14552
73.54	0.13658	73.54	0.14478
73.56	0.13637	73.56	0.14457
73.64	0.13542	73.64	0.14361
73.66	0.13516	73.66	0.14335
73.74	0.13404	73.74	0.14222

73.76	0.13373	73.76	0.14191
73.84	0.13245	73.84	0.1406
73.92	0.13104	73.92	0.13917
73.94	0.13067	73.94	0.13879
74.02	0.12912	74.02	0.1372
74.04	0.12872	74.04	0.13678
74.12	0.12703	74.12	0.13505
74.14	0.12659	74.14	0.1346
74.22	0.12478	74.22	0.13273
74.24	0.12431	74.24	0.13225
74.32	0.12239	74.32	0.13026
74.4	0.12038	74.4	0.12818
74.42	0.11987	74.42	0.12765
74.5	0.11778	74.5	0.12548
74.52	0.11724	74.52	0.12493
74.6	0.11508	74.6	0.12269
74.62	0.11453	74.62	0.12212
74.7	0.11231	74.7	0.11981
74.72	0.11175	74.72	0.11923
74.8	0.10949	74.8	0.11688
74.88	0.10721	74.88	0.1145
74.9	0.10663	74.9	0.11391
74.98	0.10434	74.98	0.11152
75	0.10377	75	0.11092
75.08	0.10147	75.08	0.10853
75.1	0.1009	75.1	0.10794
75.18	0.09863	75.18	0.10556
75.2	0.09806	75.2	0.10497
75.28	0.09581	75.28	0.10263
75.36	0.0936	75.36	0.10031
75.38	0.09305	75.38	0.09973
75.46	0.09087	75.46	0.09746
75.48	0.09034	75.48	0.0969
75.84	0.08119	75.84	0.08733
76.32	0.07076	76.32	0.07637
76.8	0.06238	76.8	0.06756
77.28	0.0558	77.28	0.06061
77.76	0.05063	77.76	0.05514
78.24	0.04654	78.24	0.05079
78.72	0.04324	78.72	0.04727
79.2	0.04051	79.2	0.04436
79.68	0.0382	79.68	0.04189
80.16	0.0362	80.16	0.03975

80.64	0.03443	80.64	0.03785
81.12	0.03284	81.12	0.03614
81.6	0.03138	81.6	0.03458
82.08	0.03004	82.08	0.03314
82.56	0.02879	82.56	0.03179
83.04	0.02761	83.04	0.03052
83.52	0.0265	83.52	0.02932
84	0.02545	84	0.02818
84.1	0.03539	84.1	0.03847
84.48	0.10774	84.48	0.11433
84.96	0.13626	84.96	0.14493
85.44	0.13753	85.44	0.14671
85.46	0.13737	85.46	0.14656
85.54	0.13662	85.54	0.14582
85.56	0.13641	85.56	0.1456
85.64	0.13546	85.64	0.14465
85.66	0.1352	85.66	0.14439
85.74	0.13408	85.74	0.14325
85.76	0.13377	85.76	0.14295
85.84	0.13249	85.84	0.14164
85.92	0.13108	85.92	0.1402
85.94	0.13071	85.94	0.13982
86.02	0.12916	86.02	0.13822
86.04	0.12876	86.04	0.1378
86.12	0.12707	86.12	0.13606
86.14	0.12663	86.14	0.13561
86.22	0.12482	86.22	0.13374
86.24	0.12435	86.24	0.13325
86.32	0.12243	86.32	0.13126
86.4	0.12042	86.4	0.12918
86.42	0.11991	86.42	0.12864
86.5	0.11782	86.5	0.12647
86.52	0.11728	86.52	0.12591
86.6	0.11512	86.6	0.12366
86.62	0.11457	86.62	0.12309
86.7	0.11235	86.7	0.12077
86.72	0.11179	86.72	0.12018
86.8	0.10952	86.8	0.11782
86.88	0.10724	86.88	0.11544
86.9	0.10667	86.9	0.11484
86.98	0.10438	86.98	0.11244
87	0.1038	87	0.11184
87.08	0.10151	87.08	0.10944

87.1	0.10094	87.1	0.10884
87.18	0.09866	87.18	0.10646
87.2	0.0981	87.2	0.10586
87.28	0.09585	87.28	0.10351
87.36	0.09363	87.36	0.10118
87.38	0.09308	87.38	0.1006
87.46	0.09091	87.46	0.09832
87.48	0.09037	87.48	0.09775
87.84	0.08122	87.84	0.08813
88.32	0.07079	88.32	0.07712
88.8	0.06241	88.8	0.06825
89.28	0.05583	89.28	0.06125
89.76	0.05066	89.76	0.05575
90.24	0.04657	90.24	0.05137
90.72	0.04326	90.72	0.04782
91.2	0.04053	91.2	0.04489
91.68	0.03822	91.68	0.0424
92.16	0.03622	92.16	0.04023
92.64	0.03445	92.64	0.03832
93.12	0.03286	93.12	0.0366
93.6	0.03141	93.6	0.03502
94.08	0.03006	94.08	0.03356
94.56	0.02881	94.56	0.0322
95.04	0.02763	95.04	0.03092
95.52	0.02652	95.52	0.02971
96	0.02547	96	0.02856
96.1	0.03541	96.1	0.03888
96.48	0.10776	96.48	0.1151
96.96	0.13628	96.96	0.14589
97.44	0.13755	97.44	0.14772
97.46	0.13739	97.46	0.14756
97.54	0.13664	97.54	0.14682
97.56	0.13642	97.56	0.14661
97.64	0.13547	97.64	0.14566
97.66	0.13521	97.66	0.1454
97.74	0.13409	97.74	0.14426
97.76	0.13379	97.76	0.14395
97.84	0.1325	97.84	0.14264
97.92	0.1311	97.92	0.1412
97.94	0.13073	97.94	0.14082
98.02	0.12918	98.02	0.13922
98.04	0.12877	98.04	0.1388
98.12	0.12708	98.12	0.13705

98.14	0.12665	98.14	0.1366
98.22	0.12483	98.22	0.13472
98.24	0.12437	98.24	0.13423
98.32	0.12244	98.32	0.13223
98.4	0.12044	98.4	0.13014
98.42	0.11992	98.42	0.12961
98.5	0.11783	98.5	0.12742
98.52	0.1173	98.52	0.12687
98.6	0.11513	98.6	0.1246
98.62	0.11458	98.62	0.12403
98.7	0.11236	98.7	0.1217
98.72	0.1118	98.72	0.12111
98.8	0.10954	98.8	0.11874
98.88	0.10726	98.88	0.11635
98.9	0.10668	98.9	0.11575
98.98	0.10439	98.98	0.11334
99	0.10382	99	0.11273
99.08	0.10152	99.08	0.11032
99.1	0.10095	99.1	0.10972
99.18	0.09868	99.18	0.10732
99.2	0.09811	99.2	0.10673
99.28	0.09586	99.28	0.10436
99.36	0.09364	99.36	0.10202
99.38	0.0931	99.38	0.10144
99.46	0.09092	99.46	0.09915
99.48	0.09039	99.48	0.09858
99.84	0.08124	99.84	0.08891
100.32	0.0708	100.32	0.07783
100.8	0.06242	100.8	0.06891
101.28	0.05584	101.28	0.06187
101.76	0.05067	101.76	0.05633
102.24	0.04658	102.24	0.05193
102.72	0.04327	102.72	0.04836
103.2	0.04054	103.2	0.0454
103.68	0.03823	103.68	0.04289
104.16	0.03623	104.16	0.04071
104.64	0.03446	104.64	0.03878
105.12	0.03287	105.12	0.03704
105.6	0.03141	105.6	0.03545
106.08	0.03007	106.08	0.03398
106.56	0.02882	106.56	0.0326
107.04	0.02764	107.04	0.03131
107.52	0.02653	107.52	0.03009

108	0.02547	108	0.02893
108.1	0.03541	108.1	0.03929
108.48	0.10777	108.48	0.11586
108.96	0.13629	108.96	0.14684
109.44	0.13756	109.44	0.14871
109.46	0.13739	109.46	0.14856
109.54	0.13664	109.54	0.14782
109.56	0.13643	109.56	0.1476
109.64	0.13548	109.64	0.14665
109.66	0.13522	109.66	0.14639
109.74	0.1341	109.74	0.14525
109.76	0.1338	109.76	0.14495
109.84	0.13251	109.84	0.14363
109.92	0.1311	109.92	0.14218
109.94	0.13073	109.94	0.1418
110.02	0.12918	110.02	0.1402
110.04	0.12878	110.04	0.13978
110.12	0.12709	110.12	0.13803
110.14	0.12665	110.14	0.13757
110.22	0.12484	110.22	0.13569
110.24	0.12437	110.24	0.1352
110.32	0.12245	110.32	0.13319
110.4	0.12044	110.4	0.1311
110.42	0.11993	110.42	0.13056
110.5	0.11784	110.5	0.12837
110.52	0.1173	110.52	0.12781
110.6	0.11514	110.6	0.12553
110.62	0.11459	110.62	0.12496
110.7	0.11237	110.7	0.12262
110.72	0.11181	110.72	0.12203
110.8	0.10954	110.8	0.11965
110.88	0.10726	110.88	0.11724
110.9	0.10669	110.9	0.11664
110.98	0.1044	110.98	0.11422
111	0.10382	111	0.11361
111.08	0.10153	111.08	0.11119
111.1	0.10096	111.1	0.11059
111.18	0.09868	111.18	0.10818
111.2	0.09812	111.2	0.10758
111.28	0.09587	111.28	0.1052
111.36	0.09365	111.36	0.10285
111.38	0.0931	111.38	0.10227
111.46	0.09093	111.46	0.09997

111.48	0.09039	111.48	0.0994
111.84	0.08124	111.84	0.08967
112.32	0.07081	112.32	0.07854
112.8	0.06243	112.8	0.06957
113.28	0.05584	113.28	0.06249
113.76	0.05068	113.76	0.05692
114.24	0.04658	114.24	0.05248
114.72	0.04328	114.72	0.04888
115.2	0.04055	115.2	0.0459
115.68	0.03823	115.68	0.04337
116.16	0.03623	116.16	0.04117
116.64	0.03446	116.64	0.03923
117.12	0.03287	117.12	0.03747
117.6	0.03142	117.6	0.03587
118.08	0.03007	118.08	0.03438
118.56	0.02882	118.56	0.033
119.04	0.02764	119.04	0.03169
119.52	0.02653	119.52	0.03046
120	0.02548	120	0.02929
120.1	0.03542	120.1	0.03969
120.48	0.10777	120.48	0.11661
120.96	0.13629	120.96	0.14778
121.44	0.13756	121.44	0.14969
121.46	0.1374	121.46	0.14954
121.54	0.13664	121.54	0.1488
121.56	0.13643	121.56	0.14859
121.64	0.13548	121.64	0.14764
121.66	0.13522	121.66	0.14738
121.74	0.1341	121.74	0.14624
121.76	0.1338	121.76	0.14593
121.84	0.13251	121.84	0.14461
121.92	0.13111	121.92	0.14316
121.94	0.13074	121.94	0.14278
122.02	0.12918	122.02	0.14118
122.04	0.12878	122.04	0.14075
122.12	0.12709	122.12	0.139
122.14	0.12665	122.14	0.13854
122.22	0.12484	122.22	0.13665
122.24	0.12437	122.24	0.13616
122.32	0.12245	122.32	0.13415
122.4	0.12044	122.4	0.13204
122.42	0.11993	122.42	0.1315
122.5	0.11784	122.5	0.1293

122.52	0.1173	122.52	0.12874
122.6	0.11514	122.6	0.12646
122.62	0.11459	122.62	0.12588
122.7	0.11237	122.7	0.12354
122.72	0.11181	122.72	0.12294
122.8	0.10955	122.8	0.12055
122.88	0.10726	122.88	0.11814
122.9	0.10669	122.9	0.11753
122.98	0.1044	122.98	0.1151
123	0.10382	123	0.11449
123.08	0.10153	123.08	0.11206
123.1	0.10096	123.1	0.11145
123.18	0.09868	123.18	0.10904
123.2	0.09812	123.2	0.10843
123.28	0.09587	123.28	0.10604
123.36	0.09365	123.36	0.10368
123.38	0.0931	123.38	0.1031
123.46	0.09093	123.46	0.10078
123.48	0.09039	123.48	0.10021
123.84	0.08124	123.84	0.09044
124.32	0.07081	124.32	0.07925
124.8	0.06243	124.8	0.07023
125.28	0.05584	125.28	0.06311
125.76	0.05068	125.76	0.05749
126.24	0.04658	126.24	0.05303
126.72	0.04328	126.72	0.04941
127.2	0.04055	127.2	0.0464
127.68	0.03824	127.68	0.04385
128.16	0.03623	128.16	0.04164
128.64	0.03446	128.64	0.03968
129.12	0.03287	129.12	0.03791
129.6	0.03142	129.6	0.03629
130.08	0.03008	130.08	0.03479
130.56	0.02882	130.56	0.03339
131.04	0.02764	131.04	0.03208
131.52	0.02653	131.52	0.03083
132	0.02548	132	0.02965
132.48	0.02447	132.48	0.02852
132.96	0.02351	132.96	0.02745
133.44	0.0226	133.44	0.02642
133.92	0.02172	133.92	0.02543
134.4	0.02088	134.4	0.02448
134.88	0.02007	134.88	0.02357

135.36	0.0193	135.36	0.02269
135.84	0.01856	135.84	0.02185
136.32	0.01785	136.32	0.02104
136.8	0.01716	136.8	0.02027
137.28	0.0165	137.28	0.01952
137.76	0.01587	137.76	0.0188
138.24	0.01526	138.24	0.0181
138.72	0.01468	138.72	0.01744
139.2	0.01412	139.2	0.01679
139.68	0.01358	139.68	0.01617
140.16	0.01306	140.16	0.01558
140.64	0.01256	140.64	0.015
141.12	0.01208	141.12	0.01445
141.6	0.01162	141.6	0.01392
142.08	0.01117	142.08	0.01341
142.56	0.01075	142.56	0.01292
143.04	0.01034	143.04	0.01244
143.52	0.00994	143.52	0.01198
144	0.00956	144	0.01154

Supplementary Table 4 Simulated pharmacokinetic parameters of dabigatran following multiple administrations of a single 150mg dose dabigatran etexilate alone or with a fore-4-dose (4 days) ticagrelor at 400mg

Dabigatran etexilate alone		Dabigatran etexilate with a fore-4-dose (4 days) ticagrelor at 400mg	
Time (h)	Dabigatran Concentration (ug/mL)	Time (h)	Dabigatran Concentration (ug/mL)
0	0	0	0
0.1	0	0.1	0
0.2	0	0.2	0
0.3	0	0.3	0
0.4	0	0.4	0
0.5	0	0.5	0
0.5773	0	0.5773	0
0.6586	0	0.6586	0
0.6773	0	0.6773	0
0.7586	0	0.7586	0
0.8	0	0.8	0
0.8586	0	0.8586	0
0.9	0	0.9	0
0.9897	0	0.9897	0
1.0679	0	1.0679	0
1.0897	0	1.0897	0
1.1679	0	1.1679	0
1.2	0	1.2	0
1.2679	0	1.2679	0
1.3	0	1.3	0
1.3679	0	1.3679	0
1.4	0	1.4	0
1.4845	0	1.4845	0
1.4915	0	1.4915	0
1.567	0	1.567	0
1.6	0	1.6	0
1.667	0	1.667	0
1.7	0	1.7	0
1.767	0	1.767	0
1.8	0	1.8	0
1.867	0	1.867	0
1.9248	0	1.9248	0
1.9794	0	1.9794	0
2	0	2	0
2.0619	0	2.0619	0

2.0794	0	2.0794	0
2.1	0	2.1	0
2.1619	0	2.1619	0
2.1794	0	2.1794	0
2.2	0	2.2	0
2.2619	0	2.2619	0
2.2794	0	2.2794	0
2.3	0	2.3	0
2.4	0	2.4	0
2.5	0	2.5	0
2.6	0	2.6	0
2.7	0	2.7	0
2.8	0	2.8	0
2.8272	0	2.8272	0
2.9	0	2.9	0
2.9691	0	2.9691	0
3.0516	0	3.0516	0
3.0691	0	3.0691	0
3.1516	0	3.1516	0
3.2	0	3.2	0
3.2516	0	3.2516	0
3.3	0	3.3	0
3.3516	0	3.3516	0
3.4	0	3.4	0
3.6	0	3.6	0
3.9702	0	3.9702	0
4	0	4	0
4.0412	0	4.0412	0
4.4	0	4.4	0
4.8	0	4.8	0
5.2	0	5.2	0
5.6	0	5.6	0
5.9381	0	5.9381	0
6	0	6	0
6.0206	0	6.0206	0
6.1031	0	6.1031	0
6.1459	0	6.1459	0
6.2031	0	6.2031	0
6.2459	0	6.2459	0
6.3031	0	6.3031	0
6.4	0	6.4	0
6.4031	0	6.4031	0
6.5	0	6.5	0

6.5031	0	6.5031	0
6.6	0	6.6	0
6.6031	0	6.6031	0
6.7	0	6.7	0
6.8	0	6.8	0
6.9	0	6.9	0
7	0	7	0
7.1	0	7.1	0
7.2	0	7.2	0
7.3	0	7.3	0
7.4	0	7.4	0
7.5	0	7.5	0
7.6	0	7.6	0
7.7	0	7.7	0
7.8	0	7.8	0
7.8382	0	7.8382	0
7.9	0	7.9	0
8	0	8	0
8.1	0	8.1	0
8.2	0	8.2	0
8.3	0	8.3	0
8.4	0	8.4	0
8.5	0	8.5	0
8.6	0	8.6	0
8.7	0	8.7	0
8.8	0	8.8	0
8.9	0	8.9	0
9	0	9	0
9.1	0	9.1	0
9.2	0	9.2	0
9.3	0	9.3	0
9.4	0	9.4	0
9.5	0	9.5	0
9.6	0	9.6	0
9.7	0	9.7	0
9.8	0	9.8	0
9.9	0	9.9	0
10	0	10	0
10.0619	0	10.0619	0
10.1	0	10.1	0
10.1619	0	10.1619	0
10.2014	0	10.2014	0
10.4	0	10.4	0

10.8	0	10.8	0
11.2	0	11.2	0
11.6	0	11.6	0
12	0	12	0
12.0412	0	12.0412	0
12.2361	0	12.2361	0
12.4	0	12.4	0
12.8	0	12.8	0
13.2	0	13.2	0
13.6	0	13.6	0
14	0	14	0
14.1506	0	14.1506	0
14.4	0	14.4	0
14.8	0	14.8	0
15.2	0	15.2	0
15.6	0	15.6	0
16	0	16	0
16.0657	0	16.0657	0
16.4	0	16.4	0
16.8	0	16.8	0
17.2	0	17.2	0
17.6	0	17.6	0
18	0	18	0
18.4	0	18.4	0
18.8	0	18.8	0
19.2	0	19.2	0
19.6	0	19.6	0
20	0	20	0
20.4	0	20.4	0
20.8	0	20.8	0
21.2	0	21.2	0
21.6	0	21.6	0
22	0	22	0
22.4	0	22.4	0
22.8	0	22.8	0
23.2	0	23.2	0
23.6	0	23.6	0
24	0	24	0
24.1	0	24.1	0
24.1615	0	24.1615	0
24.2	0	24.2	0
24.2615	0	24.2615	0
24.3	0	24.3	0

24.4	0	24.4	0
24.5	0	24.5	0
24.6	0	24.6	0
24.7	0	24.7	0
24.8	0	24.8	0
24.9	0	24.9	0
25	0	25	0
25.1	0	25.1	0
25.2	0	25.2	0
25.3	0	25.3	0
25.4	0	25.4	0
25.5	0	25.5	0
25.6	0	25.6	0
25.7	0	25.7	0
25.8	0	25.8	0
25.9	0	25.9	0
26	0	26	0
26.1	0	26.1	0
26.2	0	26.2	0
26.3	0	26.3	0
26.4	0	26.4	0
26.5	0	26.5	0
26.6	0	26.6	0
26.7	0	26.7	0
26.8	0	26.8	0
26.9	0	26.9	0
27	0	27	0
27.1	0	27.1	0
27.2	0	27.2	0
27.3	0	27.3	0
27.4	0	27.4	0
27.6	0	27.6	0
28	0	28	0
28.4	0	28.4	0
28.8	0	28.8	0
29.2	0	29.2	0
29.6	0	29.6	0
30	0	30	0
30.1	0	30.1	0
30.2	0	30.2	0
30.3	0	30.3	0
30.4	0	30.4	0
30.5	0	30.5	0

30.6	0	30.6	0
30.7	0	30.7	0
30.8	0	30.8	0
30.9	0	30.9	0
31	0	31	0
31.1	0	31.1	0
31.2	0	31.2	0
31.3	0	31.3	0
31.4	0	31.4	0
31.5	0	31.5	0
31.6	0	31.6	0
31.7	0	31.7	0
31.8	0	31.8	0
31.9	0	31.9	0
32	0	32	0
32.1	0	32.1	0
32.2	0	32.2	0
32.3	0	32.3	0
32.4	0	32.4	0
32.5	0	32.5	0
32.6	0	32.6	0
32.7	0	32.7	0
32.8	0	32.8	0
32.9	0	32.9	0
33	0	33	0
33.1	0	33.1	0
33.2	0	33.2	0
33.3	0	33.3	0
33.4	0	33.4	0
33.5	0	33.5	0
33.6	0	33.6	0
33.7	0	33.7	0
33.8	0	33.8	0
33.9	0	33.9	0
34	0	34	0
34.1	0	34.1	0
34.4	0	34.4	0
34.8	0	34.8	0
35.2	0	35.2	0
35.6	0	35.6	0
36	0	36	0
36.0828	0	36.0828	0
36.4	0	36.4	0

36.8	0	36.8	0
37.2	0	37.2	0
37.6	0	37.6	0
38	0	38	0
38.4	0	38.4	0
38.8	0	38.8	0
39.2	0	39.2	0
39.6	0	39.6	0
40	0	40	0
40.4	0	40.4	0
40.8	0	40.8	0
41.2	0	41.2	0
41.6	0	41.6	0
42	0	42	0
42.4	0	42.4	0
42.8	0	42.8	0
43.2	0	43.2	0
43.6	0	43.6	0
44	0	44	0
44.4	0	44.4	0
44.8	0	44.8	0
45.2	0	45.2	0
45.6	0	45.6	0
46	0	46	0
46.4	0	46.4	0
46.8	0	46.8	0
47.2	0	47.2	0
47.6	0	47.6	0
48	0	48	0
48.1	0	48.1	0
48.2	0	48.2	0
48.3	0	48.3	0
48.4	0	48.4	0
48.5	0	48.5	0
48.6	0	48.6	0
48.7	0	48.7	0
48.8	0	48.8	0
48.9	0	48.9	0
49	0	49	0
49.1	0	49.1	0
49.2	0	49.2	0
49.3	0	49.3	0
49.4	0	49.4	0

49.5	0	49.5	0
49.6	0	49.6	0
49.7	0	49.7	0
49.8	0	49.8	0
49.9	0	49.9	0
50	0	50	0
50.1	0	50.1	0
50.2	0	50.2	0
50.3	0	50.3	0
50.4	0	50.4	0
50.5	0	50.5	0
50.6	0	50.6	0
50.7	0	50.7	0
50.8	0	50.8	0
50.9	0	50.9	0
51	0	51	0
51.1	0	51.1	0
51.2	0	51.2	0
51.3	0	51.3	0
51.4	0	51.4	0
51.6	0	51.6	0
52	0	52	0
52.4	0	52.4	0
52.8	0	52.8	0
53.2	0	53.2	0
53.6	0	53.6	0
54	0	54	0
54.1	0	54.1	0
54.2	0	54.2	0
54.3	0	54.3	0
54.4	0	54.4	0
54.5	0	54.5	0
54.6	0	54.6	0
54.7	0	54.7	0
54.8	0	54.8	0
54.9	0	54.9	0
55	0	55	0
55.1	0	55.1	0
55.2	0	55.2	0
55.3	0	55.3	0
55.4	0	55.4	0
55.5	0	55.5	0
55.6	0	55.6	0

55.7	0	55.7	0
55.8	0	55.8	0
55.9	0	55.9	0
56	0	56	0
56.1	0	56.1	0
56.2	0	56.2	0
56.3	0	56.3	0
56.4	0	56.4	0
56.5	0	56.5	0
56.6	0	56.6	0
56.7	0	56.7	0
56.8	0	56.8	0
56.9	0	56.9	0
57	0	57	0
57.1	0	57.1	0
57.2	0	57.2	0
57.3	0	57.3	0
57.4	0	57.4	0
57.5	0	57.5	0
57.6	0	57.6	0
57.7	0	57.7	0
57.8	0	57.8	0
57.9	0	57.9	0
58	0	58	0
58.1	0	58.1	0
58.4	0	58.4	0
58.8	0	58.8	0
59.2	0	59.2	0
59.6	0	59.6	0
60	0	60	0
60.4	0	60.4	0
60.8	0	60.8	0
61.2	0	61.2	0
61.6	0	61.6	0
62	0	62	0
62.4	0	62.4	0
62.8	0	62.8	0
63.2	0	63.2	0
63.6	0	63.6	0
64	0	64	0
64.4	0	64.4	0
64.8	0	64.8	0
65.2	0	65.2	0

65.6	0	65.6	0
66	0	66	0
66.4	0	66.4	0
66.8	0	66.8	0
67.2	0	67.2	0
67.6	0	67.6	0
68	0	68	0
68.4	0	68.4	0
68.8	0	68.8	0
69.2	0	69.2	0
69.6	0	69.6	0
70	0	70	0
70.4	0	70.4	0
70.8	0	70.8	0
71.2	0	71.2	0
71.6	0	71.6	0
72	0	72	0
72.1	0	72.1	0
72.2	0	72.2	0
72.3	0	72.3	0
72.4	0	72.4	0
72.5	0	72.5	0
72.6	0	72.6	0
72.7	0	72.7	0
72.8	0	72.8	0
72.9	0	72.9	0
73	0	73	0
73.1	0	73.1	0
73.2	0	73.2	0
73.3	0	73.3	0
73.4	0	73.4	0
73.5	0	73.5	0
73.6	0	73.6	0
73.7	0	73.7	0
73.8	0	73.8	0
73.9	0	73.9	0
74	0	74	0
74.1	0	74.1	0
74.2	0	74.2	0
74.3	0	74.3	0
74.4	0	74.4	0
74.5	0	74.5	0
74.6	0	74.6	0

74.7	0	74.7	0
74.8	0	74.8	0
74.9	0	74.9	0
75	0	75	0
75.1	0	75.1	0
75.2	0	75.2	0
75.3	0	75.3	0
75.4	0	75.4	0
75.6	0	75.6	0
76	0	76	0
76.4	0	76.4	0
76.8	0	76.8	0
77.2	0	77.2	0
77.6	0	77.6	0
78	0	78	0
78.1	0	78.1	0
78.2	0	78.2	0
78.3	0	78.3	0
78.4	0	78.4	0
78.5	0	78.5	0
78.6	0	78.6	0
78.7	0	78.7	0
78.8	0	78.8	0
78.9	0	78.9	0
79	0	79	0
79.1	0	79.1	0
79.2	0	79.2	0
79.3	0	79.3	0
79.4	0	79.4	0
79.5	0	79.5	0
79.6	0	79.6	0
79.7	0	79.7	0
79.8	0	79.8	0
79.9	0	79.9	0
80	0	80	0
80.1	0	80.1	0
80.2	0	80.2	0
80.3	0	80.3	0
80.4	0	80.4	0
80.5	0	80.5	0
80.6	0	80.6	0
80.7	0	80.7	0
80.8	0	80.8	0

80.9	0	80.9	0
81	0	81	0
81.1	0	81.1	0
81.2	0	81.2	0
81.3	0	81.3	0
81.4	0	81.4	0
81.5	0	81.5	0
81.6	0	81.6	0
81.7	0	81.7	0
81.8	0	81.8	0
81.9	0	81.9	0
82	0	82	0
82.1	0	82.1	0
82.4	0	82.4	0
82.8	0	82.8	0
83.2	0	83.2	0
83.6	0	83.6	0
84	0	84	0
84.4	0	84.4	0
84.8	0	84.8	0
85.2	0	85.2	0
85.6	0	85.6	0
86	0	86	0
86.4	0	86.4	0
86.8	0	86.8	0
87.2	0	87.2	0
87.6	0	87.6	0
88	0	88	0
88.4	0	88.4	0
88.8	0	88.8	0
89.2	0	89.2	0
89.6	0	89.6	0
90	0	90	0
90.4	0	90.4	0
90.8	0	90.8	0
91.2	0	91.2	0
91.6	0	91.6	0
92	0	92	0
92.4	0	92.4	0
92.8	0	92.8	0
93.2	0	93.2	0
93.6	0	93.6	0
94	0	94	0

94.4	0	94.4	0
94.8	0	94.8	0
95.2	0	95.2	0
95.6	0	95.6	0
96	0	96	0
96.1	0.0054	96.1	0.00579
96.4	0.04671	96.4	0.05104
96.8	0.0852	96.8	0.09432
97.2	0.10293	97.2	0.11482
97.6	0.10877	97.6	0.12211
97.9	0.10854	98	0.12184
98	0.10782	98.1	0.12092
98.1	0.10682	98.2	0.11972
98.2	0.10557	98.3	0.11826
98.3	0.1041	98.4	0.11656
98.4	0.10243	98.5	0.11464
98.5	0.10057	98.6	0.11255
98.6	0.09856	98.7	0.11029
98.7	0.09642	98.8	0.1079
98.8	0.09416	98.9	0.10539
98.9	0.09182	99	0.10279
99	0.0894	99.1	0.10012
99.1	0.08693	99.2	0.09739
99.2	0.08443	99.3	0.09464
99.3	0.08192	99.4	0.09187
99.4	0.07941	99.6	0.08639
99.6	0.07444	100	0.07598
100	0.06507	100.4	0.06674
100.4	0.05678	100.8	0.05886
100.8	0.04975	101.2	0.05233
101.2	0.04394	101.6	0.04699
101.6	0.03922	102	0.04266
102	0.03541	102.1	0.04171
102.1	0.03458	102.2	0.04081
102.2	0.03379	102.3	0.03995
102.3	0.03304	102.4	0.03914
102.4	0.03233	102.5	0.03837
102.5	0.03166	102.6	0.03763
102.6	0.03102	102.7	0.03693
102.7	0.0304	102.8	0.03626
102.8	0.02982	102.9	0.03563
102.9	0.02927	103	0.03502
103	0.02874	103.1	0.03444

103.1	0.02824	103.2	0.03389
103.2	0.02776	103.3	0.03336
103.3	0.0273	103.4	0.03285
103.4	0.02686	103.5	0.03236
103.5	0.02644	103.6	0.0319
103.6	0.02604	103.7	0.03145
103.7	0.02565	103.8	0.03102
103.8	0.02528	103.9	0.0306
103.9	0.02492	104	0.0302
104	0.02458	104.1	0.02982
104.1	0.02425	104.2	0.02945
104.2	0.02393	104.3	0.02909
104.3	0.02362	104.4	0.02874
104.4	0.02332	104.5	0.02841
104.5	0.02303	104.6	0.02808
104.6	0.02275	104.7	0.02776
104.7	0.02248	104.8	0.02746
104.8	0.02222	104.9	0.02716
104.9	0.02197	105	0.02687
105	0.02172	105.1	0.02659
105.1	0.02148	105.2	0.02632
105.2	0.02124	105.3	0.02605
105.3	0.02101	105.4	0.02579
105.4	0.02079	105.5	0.02553
105.5	0.02057	105.6	0.02528
105.6	0.02036	105.7	0.02504
105.7	0.02015	105.8	0.0248
105.8	0.01995	105.9	0.02457
105.9	0.01975	106	0.02434
106	0.01955	106.1	0.02412
106.1	0.01936	106.4	0.02347
106.4	0.01881	106.8	0.02265
106.8	0.01812	107.2	0.02189
107.2	0.01747	107.6	0.02117
107.6	0.01686	108	0.02048
108	0.01628	108.4	0.01983
108.4	0.01572	108.8	0.0192
108.8	0.0152	109.2	0.0186
109.2	0.01469	109.6	0.01803
109.6	0.0142	110	0.01747
110	0.01374	110.4	0.01693
110.4	0.01329	110.8	0.01642
110.8	0.01285	111.2	0.01592

111.2	0.01244	111.6	0.01543
111.6	0.01203	112	0.01496
112	0.01164	112.4	0.01451
112.4	0.01127	112.8	0.01407
112.8	0.0109	113.2	0.01365
113.2	0.01055	113.6	0.01324
113.6	0.01021	114	0.01284
114	0.00988	114.4	0.01245
114.4	0.00957	114.8	0.01208
114.8	0.00926	115.2	0.01172
115.2	0.00896	115.6	0.01137
115.6	0.00868	116	0.01102
116	0.0084	116.4	0.01069
116.4	0.00813	116.8	0.01037
116.8	0.00787	117.2	0.01006
117.2	0.00762	117.6	0.00976
117.6	0.00737	118	0.00947
118	0.00714	118.4	0.00919
118.4	0.00691	118.8	0.00891
118.8	0.00669	119.2	0.00865
119.2	0.00648	119.6	0.00839
119.6	0.00627	120	0.00814
120	0.00607		
